# Early detection of nosocomial pathogens in air and surfaces using an innovative genetic approach for surveillance in healthcare settings

**DOI:** 10.1186/s13756-026-01725-8

**Published:** 2026-02-28

**Authors:** Antonio Martínez-Murcia, Aaron Navarro, Caridad Miró-Pina, Adrián García-Sirera, Laura Pérez, Vicente García-Román, Juan Francisco Navarro-Gracia

**Affiliations:** 1 Department of Microbiology, University Miguel Hernández, 03312 Orihuela, Alicante, Spain; 2 genetic PCR solutions®, 03300 Orihuela, Alicante, Spain; 3 Hospital Universitario de Torrevieja, 03186 Torrevieja, Alicante, Spain; 4 Hospital General Universitario de Elche, 03203 Elche, Alicante, Spain

**Keywords:** Nosocomial early warning system, Clinical air monitoring, Airborne transmission, Environmental surveillance

## Abstract

**Background:**

Healthcare-associated infections remain a major cause of morbidity, mortality, and financial burden worldwide, further exacerbated by the emergence of antimicrobial resistance. Environmental reservoirs of pathogens, including air and surfaces, play a critical role in nosocomial transmission. This study aimed to validate an integrated air and surface molecular surveillance system for the early detection of clinically relevant pathogens and resistance genes in hospital environments.

**Methods:**

Weekly air and surface samples were collected over 28 weeks from two hospitals in southeastern Spain. DNA and RNA were extracted and analysed by quantitative PCR (qPCR) targeting bacterial, fungal, and viral pathogens, as well as antimicrobial resistance genes. A subset of samples underwent shotgun metagenomic sequencing to confirm qPCR results and characterize microbial communities. Environmental findings were compared with clinical infection data from both hospitals.

**Results:**

Viral, bacterial and fungal pathogens were detected with similar patterns between air and surface samples and between hospitals. Carbapenem resistance genes showed distinct distribution profiles between hospitals. Respiratory viruses displayed strong temporal correlations with patient admissions, with viral RNA occasionally detected before clinical peaks.

**Conclusions:**

This integrated molecular surveillance system allows sensitive detection of pathogens and resistance genes in hospital environments. Coupling air and surface sampling with qPCR provides a robust tool for identifying contamination sources and tracking temporal infection trends. Its scalability and adaptability make it suitable for implementation as an early warning system in infection prevention programmes, enhancing patient safety and supporting proactive control of nosocomial infections.

**Supplementary Information:**

The online version contains supplementary material available at 10.1186/s13756-026-01725-8.

## Background

Healthcare-associated infections, also known as nosocomial infections, represent a critical challenge in hospital environments, significantly increasing patient morbidity, prolonging hospital stays, elevating healthcare costs, and contributing to the global crisis of antimicrobial resistance. It is estimated that approximately 2.6–4.3 million patients acquire a nosocomial infection in the EU/EEA each year, resulting in an estimated 37,000–91,000 deaths that can be directly attributed to these infections [1–3]. In the United States, around 687,000 nosocomial infections were reported in acute care hospitals in 2015, resulting in 72,000 associated deaths [4]. These infections significantly increase the length of hospital stays, by an average of 4 days per patient, and contribute to overcrowding by occupying an estimated 16 million additional hospital bed days in Europe each year [1]. The economic impact is equally substantial: nosocomial infections are estimated to cost European healthcare systems around €7 billion each year, while the direct cost in the United States is projected to be between $28 and $45 billion annually [1, 5]. The global burden is further exacerbated by the growing prevalence of multidrug-resistant organisms, which complicate treatment, escalate morbidity and mortality, and increase healthcare expenditure [6].

Nosocomial pathogens, especially those involved in respiratory infections, are primarily transmitted via aerosols and airborne particles, as well as through direct contact with contaminated surfaces [7–9]. Therefore, effective control of air and surface environments is crucial for the prevention of healthcare-associated infections. The present study focuses on clinically relevant pathogens, including respiratory viruses (SARS-CoV-2, Human influenza A and B viruses, and Human respiratory syncytial virus A and B) and bacterial and fungal pathogens associated with respiratory diseases (*Acinetobacter baumannii*, *Staphylococcus aureus* and *Aspergillus fumigatus*). For these pathogens, airborne transmission plays an important role. While this study places particular emphasis on airborne surveillance for respiratory pathogens, surface contamination remains highly relevant. Airborne particles may sediment onto surfaces, and many nosocomial pathogens are transmitted primarily via direct contact or the faecal-oral route. For this reason, the study also includes the non-respiratory nosocomial pathogen *Clostridioides difficile*. Due to its spore-forming capacity, *C. difficile* remains highly persistent in healthcare environments, particularly those with suboptimal decontamination practices [10].

Viral respiratory infections caused by SARS-CoV-2, Human influenza A and B viruses (HIAV, HIBV), and Human respiratory syncytial virus (HRSV) A and B are intensifying infection control challenges. SARS-CoV-2 emerged in late 2019 and rapidly spread globally, causing a pandemic. During the pandemic, the implementation of measures to limit the spread of infection such as social distancing, isolation of sick individuals, surface disinfection and air quality improvement or the use of face masks, reduced the transmission of other respiratory viruses [11]. Although the World Health Organization declared the end of the public health emergency in 2023, SARS-CoV-2 is still circulating globally together with other seasonal respiratory viruses [12, 13]. SARS-CoV-2 has been widely detected in air samples from ICUs, reinforcing the need for robust airborne pathogen monitoring systems [14, 15]. In healthcare environments, airborne transmission is particularly relevant due to aerosol-generating procedures, high patient turnover, and variable ventilation conditions, which can facilitate the accumulation and spread of infectious aerosols.

The increasing prevalence of antimicrobial resistance in the past years exacerbates the challenge of managing nosocomial infections. Indeed, antimicrobial resistance facilitates pathogen persistence in hospital settings by enabling survival under antimicrobial selective pressure [16, 17]. The importance of early detection of nosocomial pathogens is highlighted by this increased prevalence of genes associated with antimicrobial resistance. Due to this burden, the study also focused on the detection of several antimicrobial resistance genes. Notably, *Acinetobacter baumannii* frequently harbours carbapenem resistance genes such as *blaOXA-23*, *blaOXA-24*, and *blaOXA-58* [18]. Similarly, *Staphylococcus aureus* strains carrying the *mecA* gene for methicillin resistance further complicate infection control efforts [19]. Furthermore, fungal pathogens such as *Aspergillus fumigatus* are a growing threat in hospitals, particularly for immunocompromised patients. Resistance to azoles, mediated by mutations in the *cyp51A* gene, has been increasingly detected, limiting treatment options and increasing mortality rates in patients with invasive aspergillosis [14].

Environmental contamination with these pathogens highlights the dual relevance of air and surface testing in hospital settings as an early warning method with the aim of controlling the intra-hospital spread of these nosocomial pathogens. Bioaerosols and high-touch surfaces act as persistent reservoirs for these microorganisms, facilitating cross-contamination and nosocomial outbreaks [20]. Given the increasing burden of antimicrobial resistance and the continuing risk of nosocomial infections, the implementation of integrated air and surface surveillance strategies is critical for early detection and infection control. Traditional air sampling methods, such as impingers and impactors, have limitations in sensitivity and real-time applicability [21]. In contrast, advanced technologies, such as cyclonic air samplers (e.g., Coriolis^®^ µ), offer significant advantages by collecting airborne microorganisms into a liquid medium for rapid molecular diagnostics, including qPCR and next-generation sequencing [14, 21–23]. Their portability, high efficiency, and integration with genomic surveillance make them a practical tool for hospital infection control and outbreak prevention. Coupling air and surface sampling with qPCR detection enhances reliability, accuracy, sensitivity and reproducibility of pathogen detection [24–27].

This study aims to validate an integrated air and surface monitoring method developed for the detection of nosocomial pathogens and antimicrobial resistance genes in clinical environments, to demonstrate its potential to enhance infection prevention and control programmes. Here, we showed that air and surface sampling coupled with qPCR amplification allows efficient detection of antibiotic resistance-associated genes and facilitates real-time surveillance of airborne pathogens. This system could support infection prevention programmes by serving as a warning method in healthcare systems.

## Methods

### Air and surface sample collection

Air and surface samples were collected weekly from December 2023 to July 2024 in two hospitals (Hospital General Universitario de Elche and Hospital Universitario de Torrevieja) located in two cities in the province of Alicante (Spain), 45 km apart. Sampling was performed in different areas of the two hospitals according to each hospital’s policy, including Infectious Diseases unit, Emergency department and toilets. In Torrevieja, sampling was also performed in the ICU. Two independent samplings in different locations were performed in both the Infectious Diseases units and the Emergency departments of both hospitals. Air samples were collected using the Coriolis^®^ µ cyclonic air sampler (Bertin Technologies, France). This device operates by drawing air into a conical chamber where particles are separated from the air stream by centrifugal force and collected in a liquid buffer. A liquid buffer composed of DNA/RNAse free water and Triton X-100 (0.005%), was used as the collection medium. Each collection cone contained 10 mL of this buffer. The Coriolis^®^ µ was operated at a flow rate of 300 L/min for 20 min (two rounds of 10 min, as it is the device’s maximum continuous operating time). This collection time allowed the volume of the sample to be reduced to approximately 1.5 ml, thereby increasing its concentration. Samples were transported in a refrigerated container and stored at − 80 °C before DNA and RNA extraction. Surface samples were collected by wiping a GPSponge^®^ (genetic PCR solutions^®^, Orihuela, Spain) over a 40 × 40 cm area of the floor in front of the sampler. Samples were transported at room temperature, GPSponge^®^ buffer was recovered and stored at − 80 °C before DNA and RNA extraction.

### DNA and RNA extraction and purification

The genetic material of all samples was extracted and purified using the GPSpin Viral DNA/RNA kit (genetic PCR solutions^®^, Orihuela, Spain). DNA and RNA extraction and purification were performed using 200 µL of the collected samples, following the manufacturer’s instructions. Elution was performed in 50 µL. All extractions were performed in duplicate to obtain enough volume for qPCR reactions. Samples used for sequencing were extracted using the magGPS^®^ magnetic extraction system (genetic PCR solutions^®^, Orihuela, Spain) to obtain longer DNA fragments. Elution was performed in 50 µL. The DNA concentration was assessed using a Qubit fluorometer (Thermo Fisher Scientific, USA).

### PCR amplification

Pathogen detection was carried out using specific qPCR kits: AciBau qPCR (*A. baumannii*), AspFum qPCR (*A. fumigatus*), CloDif qPCR (*C. difficile*), and StaAur qPCR (*S. aureus*). Viral respiratory pathogens (SARS-CoV-2, HIAV, HIBV, HRSV-A/B) were detected with the HVRD RT-qPCR Multiplex kit. Antimicrobial resistance determinants were identified with the MRG qPCR (*mecA*), CR-blaOXA-23 qPCR, CR-blaOXA-24 qPCR, CR-blaOXA-58 qPCR, and AR-cyp51A qPCR kits. All qPCR kits above were obtained from genetic PCR solutions^®^, Orihuela, Spain. MRG qPCR assays were performed on StaAur-positive samples; CR-blaOXA-23 qPCR, CR-blaOXA-24 qPCR and CR-blaOXA-58 qPCR assays on AciBau-positive samples and AR-cyp51A qPCR assays on AspFum-positive samples. Reactions were prepared according to manufacturer’s instructions, using 5 µL of sample per reaction. Amplification was performed on a StepOnePlus^TM^ Real-Time PCR System (Applied Biosystems, USA) or a CFX96 Real-Time PCR System (Bio-Rad, USA). The cycling protocol included an initial activation step at 95 °C for 2 min, followed by 40 amplification cycles including denaturation at 95 °C for 5 s, annealing/extension at 60 °C for 20 s, and data collection at the end of each cycle. For RNA targets, an initial retrotranscription step at 50 °C for 10 min was added. Positive control reactions using the Standard Template (genetic PCR solutions^®^, Orihuela, Spain) and negative control reactions with nuclease-free water, were included in every qPCR run. An Internal Control (IC) reaction targeting an exogenous DNA sequence was included in all reactions to monitor PCR inhibition. The IC is optimized to detect inhibitors without affecting the sensitivity towards the pathogen target. Fluorescence signals were acquired in the FAM channel for the main target and HEX channel for the IC in singleplex assays. For the HVRD RT-qPCR Multiplex assay, the fluorogenic signal for SARS-CoV-2 was collected in the FAM channel, Cy5.5 was used for HIAV, Cy5 for HIBV, Texas Red-X for HRSV-A/B, and the HEX channel for the IC. Calibration curves were generated with 10-fold serial dilutions of the Standard Template (10⁶–10 copies).

### Metagenomic sequencing and comparative sequence analysis

Shotgun metagenomic sequencing was performed on a subset of 12 samples (6 surface and 6 air samples). Libraries were prepared with the Ligation Sequencing Kit V14, SQK-LSK114 (Oxford Nanopore Technologies, UK), following the manufacturer’s protocol. Sequencing was conducted on the MinION Mk1C device (Oxford Nanopore Technologies, UK) using a R10.4.1 Flongle Flow Cell (Oxford Nanopore Technologies, UK) for each sample. Raw FAST5 sequencing data were basecalled with Dorado software (Oxford Nanopore Technologies, UK). BAM files were converted to FASTQ using SAMtools. Quality control was performed with NanoStat, followed by filtering with NanoFilt to retain reads with a Phred quality score ≥ 15. Taxonomic classification of filtered reads was conducted with Centrifuge software v1.0.4 (Johns Hopkins University, USA) using the p_compressed + h+v database (prokaryotic, human, viral). Functional annotation of the reads was performed with DIAMOND (University of Tübingen, Germany) against the NCBI non-redundant protein database, and results were visualized with MEGAN6 (University of Tübingen, Germany).

## Results

### Detection of bacterial and fungal pathogens, and genes coding for antibiotic resistances

The presence of several bacterial pathogens, fungi and resistance genes potentially involved in nosocomial infections was assessed by qPCR in air and surface samples collected from different areas of two hospitals (Hospital General Universitario de Elche and Hospital Universitario de Torrevieja), as described in the Methods section. Additionally, whenever available, clinical data were obtained on the number of patients admitted with infections caused by these bacteria and fungi together with the associated antibiotic resistance profiles during the study period.

**Fig. 1:**
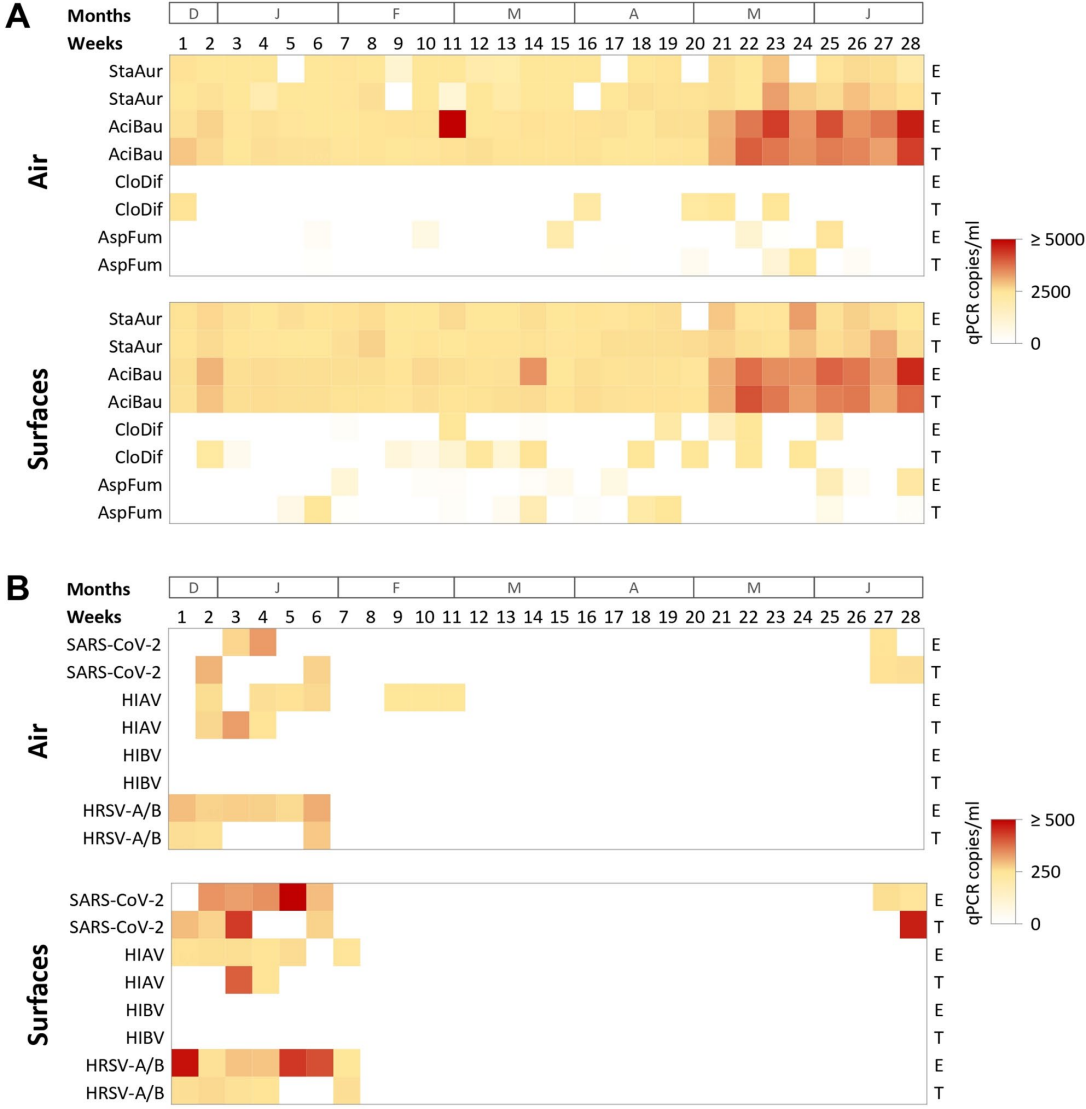
Temporal distribution of bacterial and viral qPCR detections in air and surface samples

**Fig. 2:**
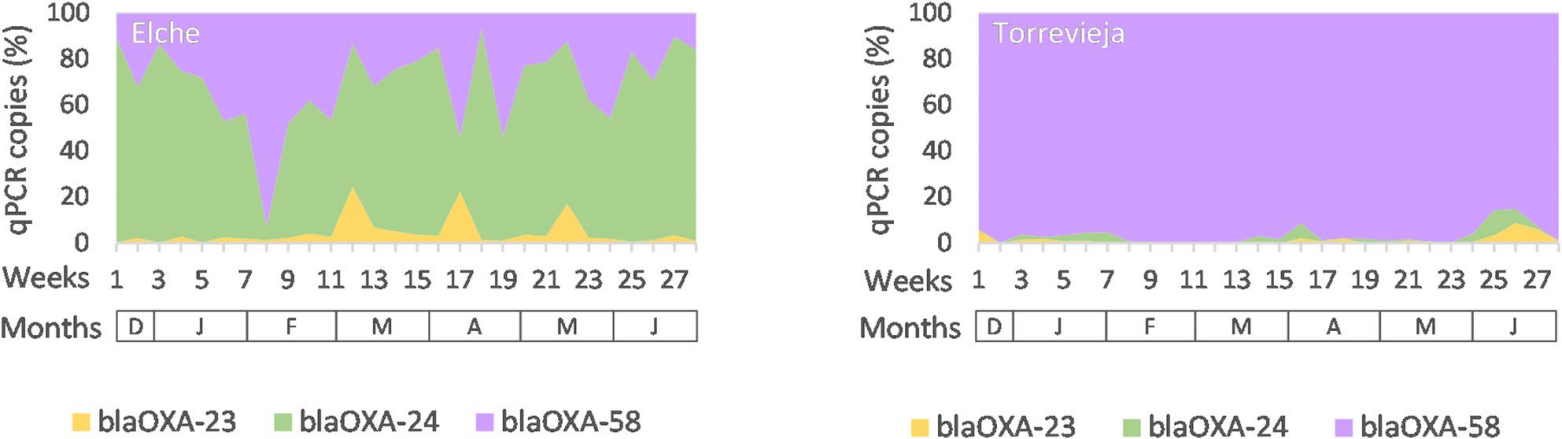
Relative abundance of carbapenem-resistance genes (*blaOXA*)

*Aspergillus fumigatus* was detected sporadically in both surface and air samples, generally at low qPCR copy numbers (Fig. 1A). Cases of *A. fumigatus* infection were not reported in either hospital during the same period in any of the hospitals (Additional file 1A). The *cyp51A*-TR_34_ insertion, associated with azole resistance in *A. fumigatus*, was not detected in any *A. fumigatus*-positive sample. *C. difficile* was detected sporadically throughout the sampling period, primarily in surface samples, although occasionally also in air samples (Fig. 1A). Detected copy numbers were generally low (Additional file 1B). However, patients with *C. difficile* infections were admitted in most weeks throughout the sampling period in both hospitals (Additional file 1B). *S. aureus* was consistently detected in approximately half of the collected samples, including both air and surfaces, and across the entire study period (Fig. 1A). Copy numbers were typically higher during the spring–summer months (from week 21 onwards) (Fig. 1A). Clinical monitoring in both hospitals focused exclusively on patients infected with methicillin-resistant *S. aureus* (MRSA). MRSA cases were reported in most weeks during the sampling period, but their frequency did not match the pattern of qPCR environmental detection (Additional file 1 C). The *mecA* gene, associated with methicillin resistance in *S. aureus*, was detected in the majority of *S. aureus*-positive samples and was frequently present at higher copy numbers than the bacterial DNA itself (Additional file 1D). *A. baumannii* was detected in most air and surface samples throughout the entire sampling period, however, only one patient with an *A. baumannii* infection was reported in Elche during the same period (Additional file 1E). From week 21 (07/05/2024) onwards, the copy numbers of *A. baumannii* DNA increased by approximately one logarithm in both hospitals (Fig. 1A and Additional file 1E). Resistance genes *blaOXA-23*, *blaOXA-24/40*, and *blaOXA-58*, associated with carbapenem resistance in *A. baumannii*, were also detected in *A. baumannii*-positive samples throughout the study, but their copy number did not increase from week 21 (Additional file 1 F–H). Notably, the distribution of these genes differed between hospitals: in Elche, *blaOXA-24/40* was predominant (65.0%), followed by *blaOXA-58* (30.6%) and *blaOXA-23* (4.4%); while in Torrevieja, *blaOXA-58* was overwhelmingly prevalent (96.7%), with only minor detection of *blaOXA-24/40* (1.9%) and *blaOXA-23* (1.4%) (Fig. 2). In Elche, a higher qPCR copy number of *blaOXA-58* was detected in week 8, coinciding with the only week in which a patient with *A. baumanni* infection was admitted (Fig. 2). Taken together, these results demonstrate that a broad range of bacterial and fungal pathogens, as well as antimicrobial resistance genes, could be consistently detected in air and surface samples. However, a direct correlation between environmental detection and the number of admitted patients the same week was not always evident.

Hot plots representing the weekly average qPCR copy numbers (copies/mL) of (A) bacterial and fungal targets: *S. aureus* (StaAur), *A. baumannii* (AciBau), *C. difficile* (CloDif), and *A. fumigatus* (AspFum); and (B) viral targets: SARS-CoV-2, HIAV, HIBV, and HRSV-A/B, detected in air and surface samples from Hospital General Universitario de Elche (E) and Hospital Universitario de Torrevieja (T). Each cell represents the average qPCR copy number detected across all sampled areas within each hospital during the corresponding week. Colour intensity reflects relative abundance according to the scale bars shown. Weeks and corresponding months (December–June) are indicated above.

Weekly proportion (%) of qPCR-positive detections for *blaOXA-23*, *blaOXA-24/40*, and *blaOXA-58* genes in air and surface samples collected from Hospital General Universitario de Elche and Hospital Universitario de Torrevieja. The relative abundance was calculated as the percentage of each *blaOXA* gene copy number with respect to the total sum of *blaOXA-23*, *blaOXA-24/40*, and *blaOXA-58* copy numbers detected each week across all sampled areas. The x-axis represents weeks and corresponding months (December–June).

### Metagenomic sequencing

Shotgun metagenomic DNA sequencing was performed on a subset of 12 samples collected at Torrevieja Hospital (six surface and six air samples from different areas). This untargeted approach enabled comprehensive taxonomic and functional profiling of microbial communities. To minimize the bias due to variable DNA input, air samples were selected based on bacterial load prior to sequencing. Sequencing of surface samples yielded an average of 39,079 reads per sample, whereas air samples averaged 34,441 reads. Quality control using NanoStat, followed by filtering with NanoFilt, retained 46.4% of surface and 45.6% of air reads with a Phred quality score ≥ 15. Taxonomic classification (Centrifuge v1.0.4) confirmed the presence of *Staphylococcus* and *Acinetobacter* in the analysed surface samples, consistent with qPCR findings. Additional clinically relevant genera, including *Escherichia*, *Pseudomonas*, and *Clostridium*, were also identified in both surface and air samples. Microbial diversity analyses revealed marked differences between sample types. In surface samples, *Escherichia* was the most abundant genus, whereas *Paraburkholderia* predominated in air samples. Notably, *Paraburkholderia* was not identified in any surface sample.

### Detection of respiratory viruses

**Fig. 3:**
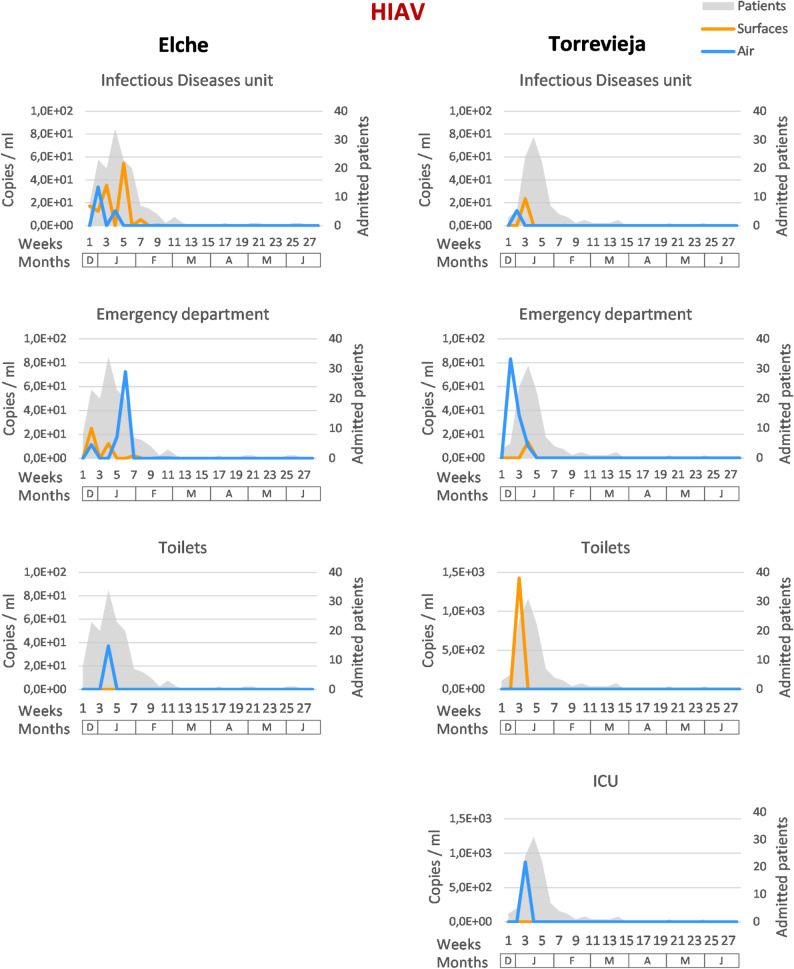
HIAV qPCR detection and hospitalised patients with HIAV infection

Respiratory viruses, including SARS-CoV-2, HIAV, HIBV, and HRSV-A/B, were also monitored weekly in air and surface samples collected from both hospitals during the study period. Data on the number of patients hospitalised with respiratory infections caused by these viruses were also provided, allowing comparison between environmental detection and clinical incidence. HRSV-A/B RNA was detected in air and surface samples from both hospitals, mainly during the winter months (weeks 1–13), coinciding with periods of patient admissions due to HRSV infections. In the Hospital General Universitario de Elche, the maximum number of patients admitted with HRSV-A/B infection was approximately five times higher than in the Hospital Universitario de Torrevieja. This increased clinical incidence correlated with higher qPCR copy numbers detected in environmental samples, particularly in those collected from the Infectious Diseases unit (Fig. 1B and Additional file 1I). In Torrevieja, although the number of hospitalised patients with HRSV-A/B infection was lower, viral RNA was still detected in both air and surface samples during the same period (Fig. 1B). HIBV was not detected in any air or surface sample from either hospital (Fig. 1B). Only one patient with HIBV infection was admitted to the Hospital General Universitario de Elche during the first two weeks of the sampling period, which is consistent with the absence of environmental detections. In the case of HIAV, viral RNA was detected in several air and surface samples from both hospitals, primarily during the winter season (weeks 1–13) (Fig. 1B). Detection occurred mainly between weeks 2 and 6 in Elche and weeks 2 and 4 in Torrevieja, coinciding with the epidemiological window in which most patients were admitted with HIAV infection (Fig. 3). In several instances, HIAV RNA was detected in environmental samples before the number of hospitalised patients reached its maximum, (Fig. 3). SARS-CoV-2 RNA was also detected in air and surface samples collected from both hospitals (Fig. 1B). Two distinct waves of COVID-19 patient admissions were recorded during the study period. The first wave, occurring in winter, included more than ten patients admitted during weeks 2 to 5 in Elche and weeks 2 to 6 in Torrevieja. The second wave, recorded in spring–summer, included more than ten patients admitted in weeks 26 to 27 in Elche and nine patients in weeks 27 to 28 in Torrevieja (Additional file 1 J). SARS-CoV-2 RNA was detected in several environmental samples during both waves, generally with higher copy numbers during the first (Fig. 1B). In multiple cases, the detection of SARS-CoV-2 RNA in air and surface samples preceded the peak in hospital admissions (Additional file 1 J).

Weekly qPCR copy numbers of HIAV RNA (copies/mL) detected in air (blue) and surface (orange) samples collected from different hospital areas (Intensive Care Unit (ICU), toilets, Emergency Department, and Infectious Diseases unit) at Hospital General Universitario de Elche and Hospital Universitario de Torrevieja between weeks 1 and 28. Data from the Infectious Diseases unit and the Emergency Department represent the mean of two independent samplings performed in different areas. The number of patients admitted with HIAV infection (grey) is shown for comparison. The x-axis represents weeks and corresponding months (December–June), and the y-axes indicate qPCR copy numbers and number of admitted patients.

## Discussion

Control of environmental pathogen contamination in clinical environments is of utmost importance for the control of nosocomial infections. Here, we have validated a method for air and surface monitoring for the detection of nosocomial pathogens and antimicrobial resistance genes in hospital settings. qPCR analysis allowed detection of selected nosocomial pathogens including respiratory viruses, bacteria, fungi and their antimicrobial resistance genes. The system was applied continuously over several months, allowing the evaluation of temporal patterns and their relationship with clinical data. Furthermore, shotgun metagenomics were performed on air and surface samples, confirming qPCR results and supporting the system’s potential to monitor environmental pathogens.

With the exception of HIBV, all targeted pathogens in this study were detected in both air and surface samples using the qPCR-based monitoring system over a 28-week period. Overall, detection patterns for bacteria, fungi, and viruses were comparable between air and surface samples, as well as between the two hospitals, which are located in different cities approximately 45 km apart. The qPCR data were compared with the number of patients admitted to both hospitals during the same period who were diagnosed with the corresponding infections. For bacterial and fungal DNA, environmental detection did not always correlate with the number of hospitalised patients infected with the same pathogens at corresponding dates. *A. fumigatus* sporadic qPCR detection in both air and surface samples was consistent with the absence of patients admitted with *A. fumigatus* infection during the study period. *C. difficile* was detected more frequently in surface samples than in air samples, consistent with its predominant transmission via the faecal-oral route. However, its detection in some air samples suggests possible aerosolization or resuspension of spores or DNA from contaminated surfaces. Elevated *A. baumannii* levels were detected in both hospitals and in both air and surface samples from mid-spring through early summer (from week 21 onwards), coinciding with the previously reported seasonal rise in *A. baumannii* infection rates during warmer months [28]. However, no clinical cases were reported in either hospital during this period. Methicillin-resistant *S. aureus* infections were significantly more frequent, but periods with higher numbers of admitted patients did not always coincide with positive environmental detections of *S. aureus* and *mecA*. Interestingly, an increase in *S. aureus* and *mecA* qPCR copy numbers detected between weeks 21 and 25 was followed by a noticeable rise in MRSA clinical cases at both hospitals. In contrast, during weeks 5 to 9 in Torrevieja, the rise of MRSA infections did not translate in a significant increase in environmental DNA detection. Taken together, these findings suggest that molecular environmental monitoring could serve as a valuable tool to assess and mitigate contamination risks associated with *A. baumannii* and MRSA in hospital settings. These pathogens may persist in the hospital environment independently of ongoing outbreaks, as observed in previous studies [29, 30]. It is plausible that environmental contamination reflects a latent reservoir of pathogens that may contribute to sporadic transmission events or increase infection risk under specific conditions. Nevertheless, the consistent detection of specific organisms underscores the need for continuous environmental surveillance, as their presence may precede or persist beyond clinical cases, acting as a silent indicator of potential risk.

Several antimicrobial resistance genes were also detected by qPCR. The *mecA* gene, associated with methicillin resistance in *S. aureus*, was generally detected at higher copy numbers than *S. aureus* itself in *S. aureus* positive samples. This discrepancy likely reflects the presence of multiple *mecA* copies in certain *S. aureus* strains. Indeed, although *mecA* is generally found in a single copy in *S. aureus* genome, multiple copies have been reported in some coagulase-negative *Staphylococcus* isolates [31]. In addition, highly homologous *mecA* genes have been identified in other *Staphylococcus* species [32, 33], which could also contribute to the higher qPCR signal observed. qPCR analysis further revealed distinct profiles of carbapenem resistance genes associated with *A. baumannii* between the two hospitals. Although all three *blaOXA* variants were detected in both hospitals in air and surface samples, their relative abundance differed. In Elche, *blaOXA-24/40* predominated, whereas *blaOXA-58* was most frequently detected in Torrevieja. These results are consistent with previous studies showing that *blaOXA-23* is the most globally widespread variant, while *blaOXA-24/40* and *blaOXA-58* are more prevalent in some regions, including Spain [34, 35], . Our findings indicated that *blaOXA* gene distribution can vary substantially even between hospitals within the same geographic area. These observations may reflect differences in antimicrobial usage patterns, patient populations, or clonal distribution of environmental strains within each facility. Interestingly, in Elche, *blaOXA* gene distribution varied markedly in week 8, when a pronounced increase in blaOXA-58 copy number was observed, largely due to its high detection level in the surface sample from the toilet area. During that same week, a patient infected with *A. baumannii* was admitted. It is plausible that this patient carried an *A. baumannii* strain harbouring the *blaOXA-58* gene, and that environmental contamination originated from this source. Given that *blaOXA-58* predominated in Torrevieja, the strain might have circulated between the two hospitals. The *cyp51A*-TR_34_ insertion, associated with azole resistance, was not detected, consistent with the low environmental abundance of its host. The detection of resistance genes in environmental samples, even in the absence of active infections, suggests a stable persistence of resistant genetic elements within the hospital microbiome. These results underscore the importance of environmental surveillance not only for pathogen detection but also for monitoring selective pressures and resistance dynamics within healthcare infrastructures.

The system was also applied to detect respiratory viruses (SARS-CoV-2, HIAV, HIBV, and HRSV-A/B), providing a complementary perspective on airborne and surface contamination. Unlike bacterial and fungal targets, respiratory virus detections showed a clear temporal correlation with periods of increased patient admissions. HIBV was not detected in any sample, consistent with the very low number of hospitalised patients infected with this virus and with the almost complete absence of its circulation during the study period. Detection of the remaining respiratory viruses was restricted to the weeks when patients were admitted with respiratory viral infections, predominantly during winter. SARS-CoV-2 was also detected during the final two weeks of sampling, coinciding with an increase of COVID-19 admissions. Overall, the temporal patterns of viral RNA detection in environmental samples mirrored the distribution of clinical cases, reflecting real epidemiological trends and suggesting that air and surface sampling can serve as an effective tool for real-time monitoring of viral transmission dynamics in hospital environments. In several instances, viral RNA was detected in air and surface samples before the peak in hospitalised cases, particularly during the winter waves of COVID-19 and influenza. Conversely, in some weeks, viral RNA was detected after the clinical peaks, possibly reflecting environmental persistence or to continued viral shedding by hospitalised patients. This temporal alignment supports the potential of environmental air and surface monitoring as an early indicator of emerging outbreaks. However, additional data are required to establish a robust correlation. These findings are consistent with previous studies reporting that bioaerosol sampling can effectively detect early viral circulation in healthcare settings [36, 37].

Shotgun metagenomic DNA sequencing was performed on a subset of air and surface samples from Torrevieja Hospital as a proof-of-concept to validate the sequencing protocol. Taxonomic classification confirmed the presence of *Staphylococcus* and *Acinetobacter*, consistent with qPCR data, and additionally revealed other clinically relevant genera such as *Escherichia*, *Pseudomonas*, and *Clostridium*. The sequencing data provided a preliminary overview of the environmental microbiota and suggested differences in microbial composition between air and surface samples. *Escherichia* was the most abundant genus in surface samples, whereas *Paraburkholderia* was detected predominantly in air samples but was not detected in surface samples. The predominance of *Escherichia* on surfaces agrees with previous reports describing its persistence in healthcare environments [38, 39]. Likewise, the presence of *Burkholderia-Paraburkholderia* species in bioaerosol samples has also been documented in earlier studies [40, 41]. These observations are consistent with the concept that distinct ecological niches contribute to differentiation of microbial communities across environmental matrices [42]. While qPCR enables the fast detection of specific pathogens, non-targeted approaches such as metagenomic sequencing offer the potential to detect a broader range of microorganisms [43]. In addition, functional annotation of metagenomic reads may provide opportunities to explore resistance and virulence factors beyond those included in the qPCR panel. However, the limited amount of environmental DNA material available in air and surface samples, together with DNA fragmentation, resulted in limited sequencing depth. These factors represent important limitations in the metagenomic analyses of this study, and therefore these results should be interpreted as exploratory. Future work could focus on further optimizing air and surface sampling protocols for shotgun metagenomic sequencing to establish a sequencing-based surveillance system.

The integrated molecular surveillance approach proved effective, confirming that environmental DNA analysis can complement traditional clinical surveillance by revealing patterns of contamination and persistence. However, several limitations of the study must be acknowledged. First, the lack of detailed clinical data, such as resistance profiles, limited the ability of the study to establish clear, direct links between environmental and clinical cases. Additionally, clinical information was limited to the hospitalised patients, excluding data from healthcare workers and visitors who may also contribute to environmental contamination and thus influence detection results. Moreover, qPCR-based detection captures DNA from both viable and non-viable organisms, which may lead to an overestimation of infection risk in the absence of viability assessment. Many detections were close to the assay’s limit of detection, and the biological relevance of these low-level signals remains uncertain. Future studies should incorporate larger datasets to evaluate the clinical and epidemiological significance of low qPCR copy number detections, ideally in combination with viability assays or culture-based confirmation. Despite these limitations, the trends observed were consistent across the data analysed, supporting the robustness and reliability of the findings.

The results of this study support the implementation of integrated molecular surveillance strategies in healthcare settings, combining environmental sampling with highly sensitive qPCR assays. This approach enables near real-time monitoring of pathogen presence and resistance profiles, providing actionable data for infection control teams. Moreover, the qPCR analysis can be easily expanded to detect additional targets, including other pathogens, antimicrobial resistance determinants, and virulence-associated genes. Its adaptability, scalability, and non-invasive nature make it suitable for routine use across diverse hospital environments. In practice, the optimal sampling frequency and deployment strategy should be adapted to the local infection prevention framework, risk level, and available resources. A risk-based approach could prioritize high-risk areas and increase sampling frequency during periods of higher clinical incidence or suspected outbreaks. Incorporating such surveillance systems into standard infection prevention protocols could facilitate data-driven decisions on the selection and implementation of preventive measures. Ultimately, this strategy provides a proactive framework for enhancing surveillance practices, improving patient safety, and mitigating the impact of nosocomial infections.

## Conclusions

This study shows that the combined use of high-efficiency cyclonic air sampling and surface sampling, followed by qPCR, enables the reliable detection of clinically relevant nosocomial pathogens even at low environmental concentrations. Detection of specific targets by qPCR was supported by sequencing, reinforcing the validity of the obtained results. Importantly, the method is not limited to a predefined set of pathogens. It can be adapted to detect virtually any microorganism, antimicrobial resistance gene or virulence factor of interest through appropriate primer design (qPCR). This versatility allows its implementation for targeted surveillance programmes in clinical settings, contributing to early warning systems and guiding cleaning protocols before clinical cases emerge.

## Supplementary Information

Below is the link to the electronic supplementary material.

Supplementary Material 1

## Data Availability

The datasets used and/or analysed during the current study are available from the corresponding author on reasonable request.

## Abbreviations

qPCR: quantitative PCR
DNA: Deoxyribonucleic acid
RNA: Ribonucleic acid
EU/EEA: European union / European economic area
SARS-CoV-2: Severe acute respiratory syndrome coronavirus 2
HIAV: Human influenza A virus
HIBV: Human influenza B virus
HRSV: Human respiratory syncytial virus
MRG: methicillin resistance gene
MRSA: methicillin-resistant Staphylococcus aureus
ICU: Intensive care unit
