# Supplementary material for: Early detection of nosocomial pathogens in air and surfaces using an innovative genetic approach for surveillance in healthcare settings
Source: Antimicrob Resist Infect Control. 2026 Feb 28;15:51. doi: 10.1186/s13756-026-01725-8 (PMC13059197; doi:10.1186/s13756-026-01725-8)
Supplement: Supplementary file 1 — Supplementary Material 1 [file 13756_2026_1725_MOESM1_ESM.pdf]

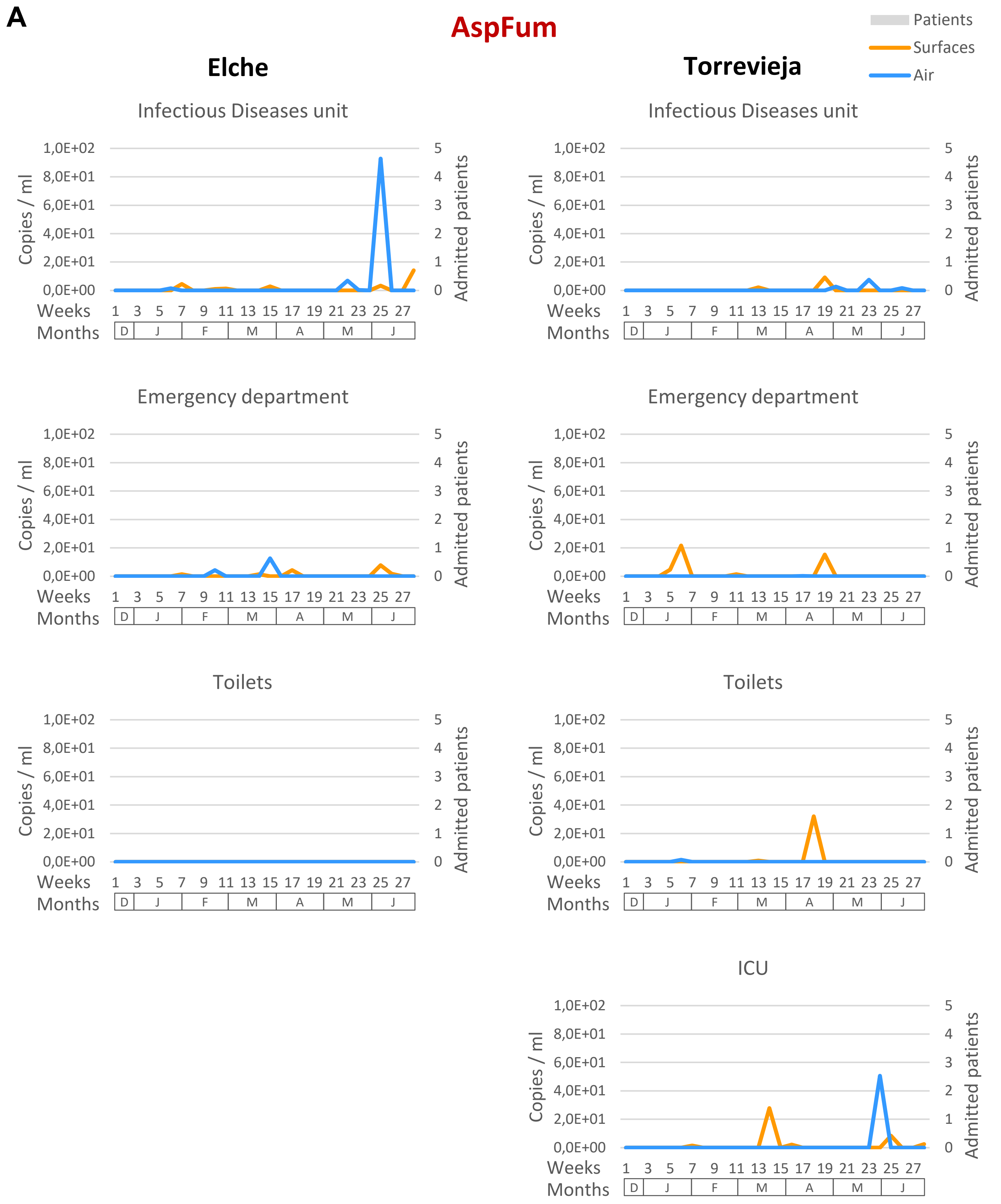
A
AspFum
Patients
Surfaces
Air
Elche
Torrevieja
Infectious Diseases unit
Emergency department
Toilets
ICU
Copies / ml
Admitted patients
1,0E+02
8,0E+01
6,0E+01
4,0E+01
2,0E+01
0,0E+00
5
4
3
2
1
0
Weeks
1 3 5 7 9 11 13 15 17 19 21 23 25 27
Months
D J F M A M J

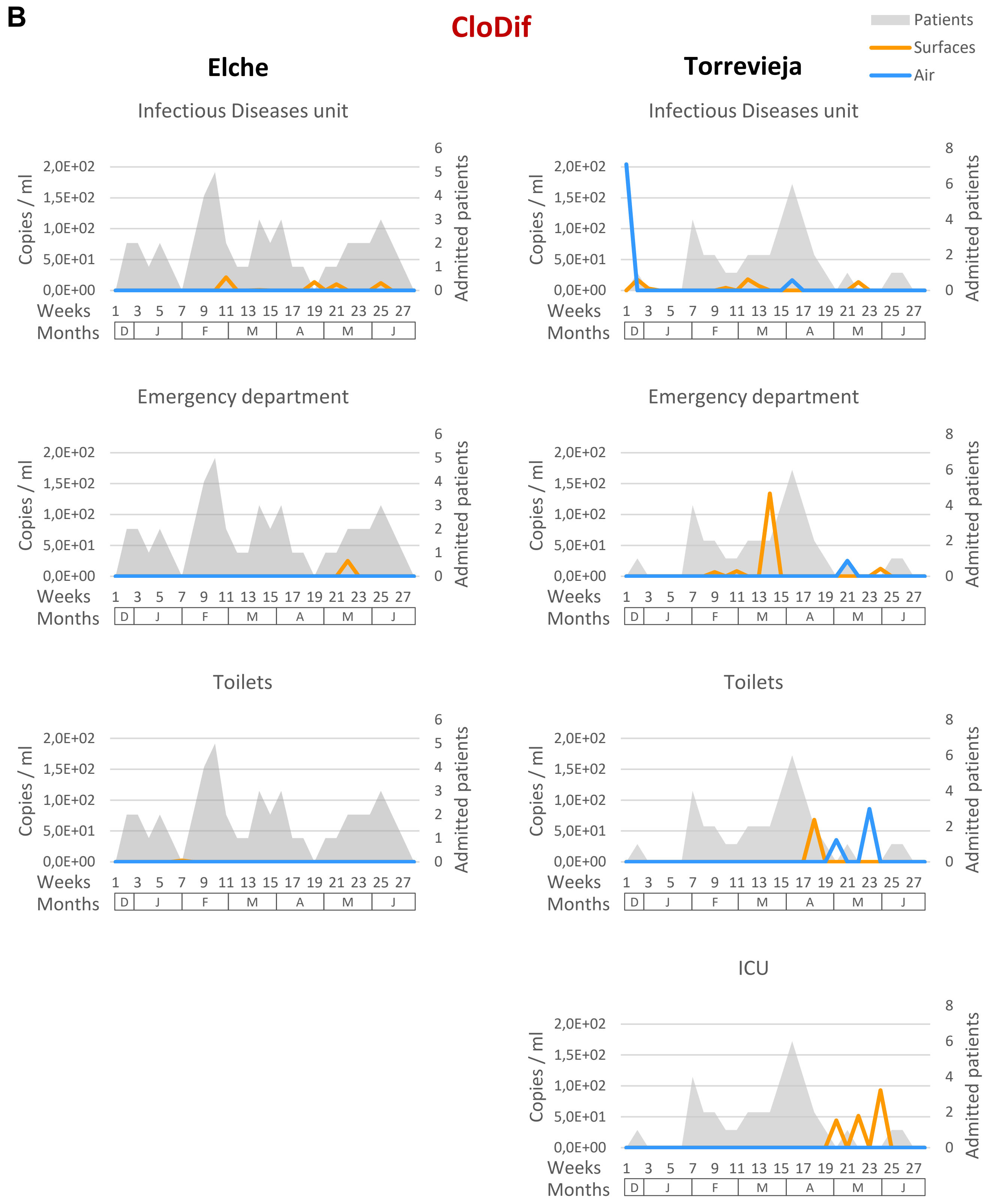
B
CloDif
Patients
Surfaces
Air
Elche
Torrevieja
Infectious Diseases unit
Emergency department
Toilets
ICU
Copies / ml
Admitted patients
2,0E+02
1,5E+02
1,0E+02
5,0E+01
0,0E+00
Weeks
1 3 5 7 9 11 13 15 17 19 21 23 25 27
Months
D J F M A M J

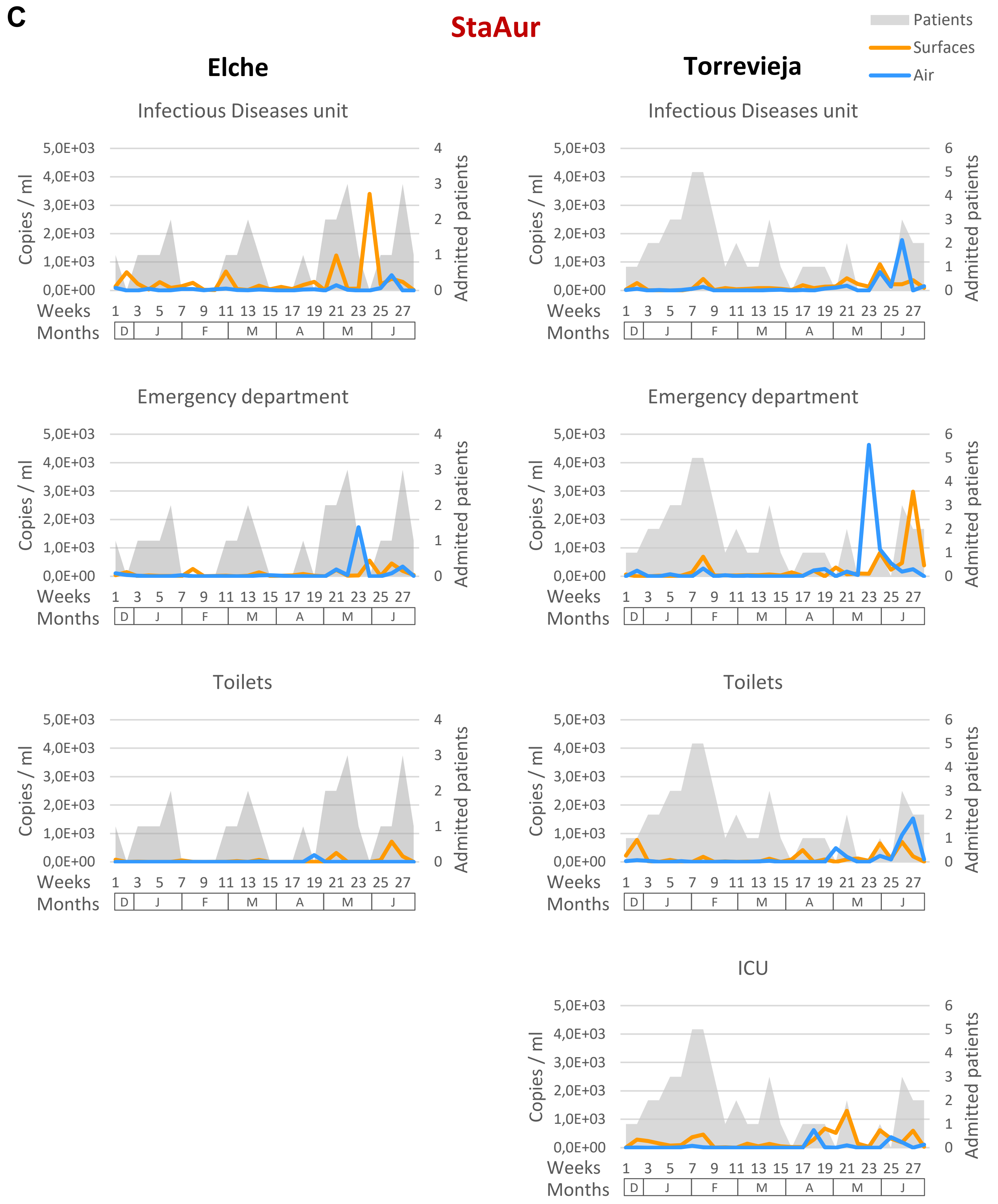
C
StaAur
Patients
Surfaces
Air
Elche
Torrevieja
Infectious Diseases unit
Emergency department
Toilets
ICU
Copies / ml
Admitted patients
Weeks
Months
D J F M A M J

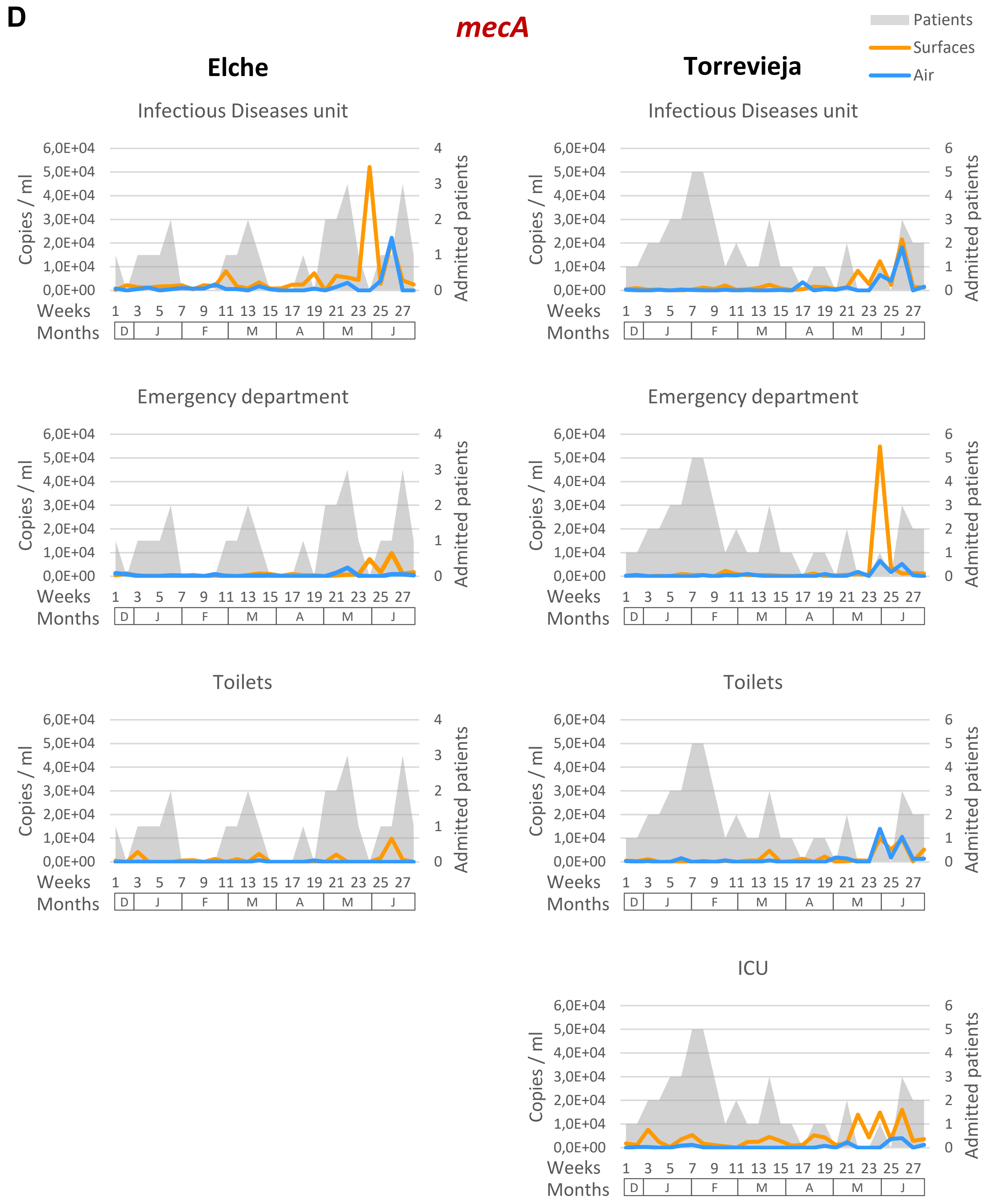
D
mecA
Elche
Torrevieja
Patients
Surfaces
Air
Infectious Diseases unit
Emergency department
Toilets
ICU
Copies / ml
Admitted patients
Weeks
Months
6,0E+04
5,0E+04
4,0E+04
3,0E+04
2,0E+04
1,0E+04
0,0E+00
1 3 5 7 9 11 13 15 17 19 21 23 25 27
D J F M A M J

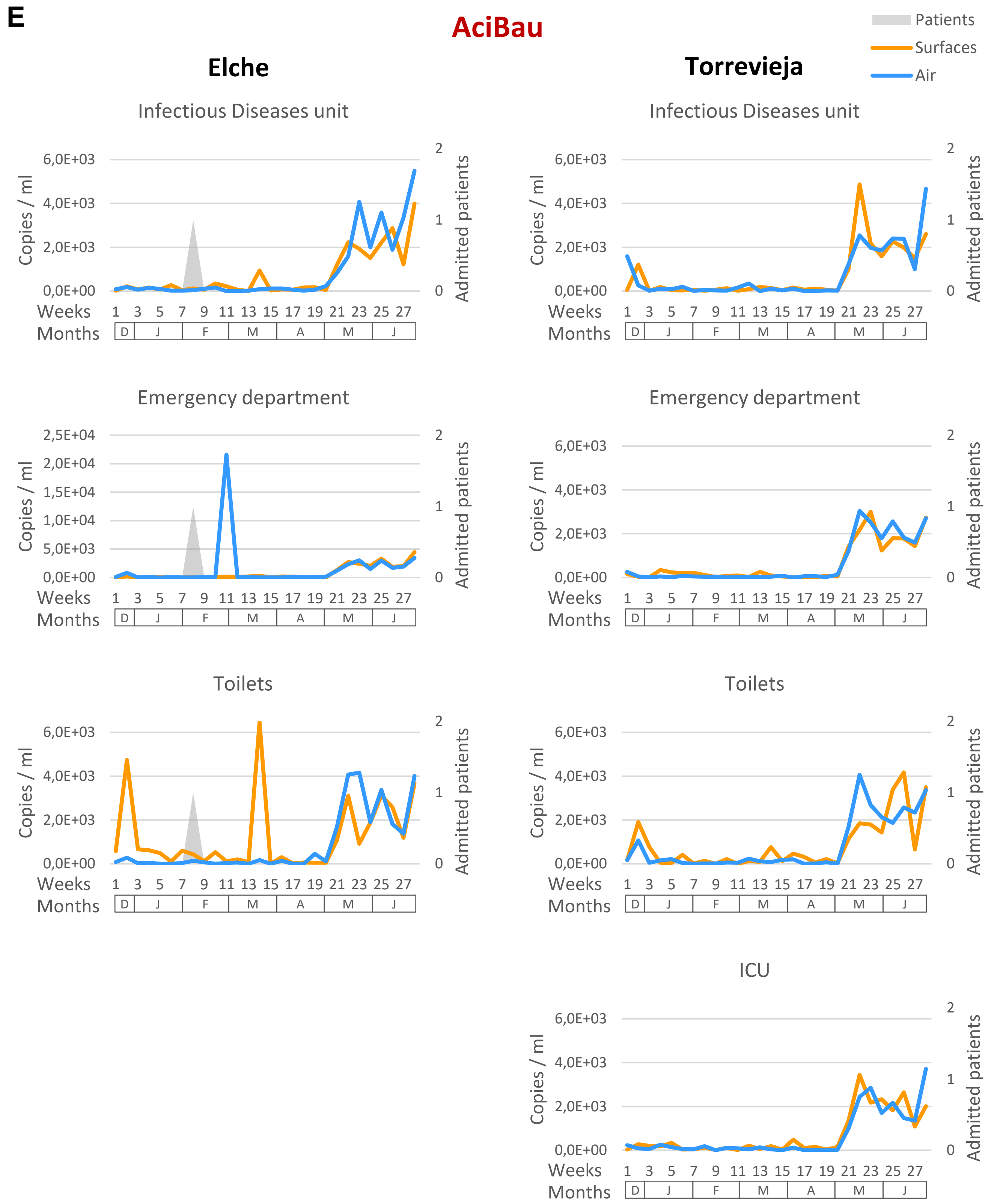
E
AciBau
Patients
Surfaces
Air
Elche
Torrevieja
Infectious Diseases unit
Emergency department
Toilets
ICU
Copies / ml
Admitted patients
6,0E+03
4,0E+03
2,0E+03
0,0E+00
2,5E+04
2,0E+04
1,5E+04
1,0E+04
5,0E+03
Weeks
1 3 5 7 9 11 13 15 17 19 21 23 25 27
Months
D J F M A M J

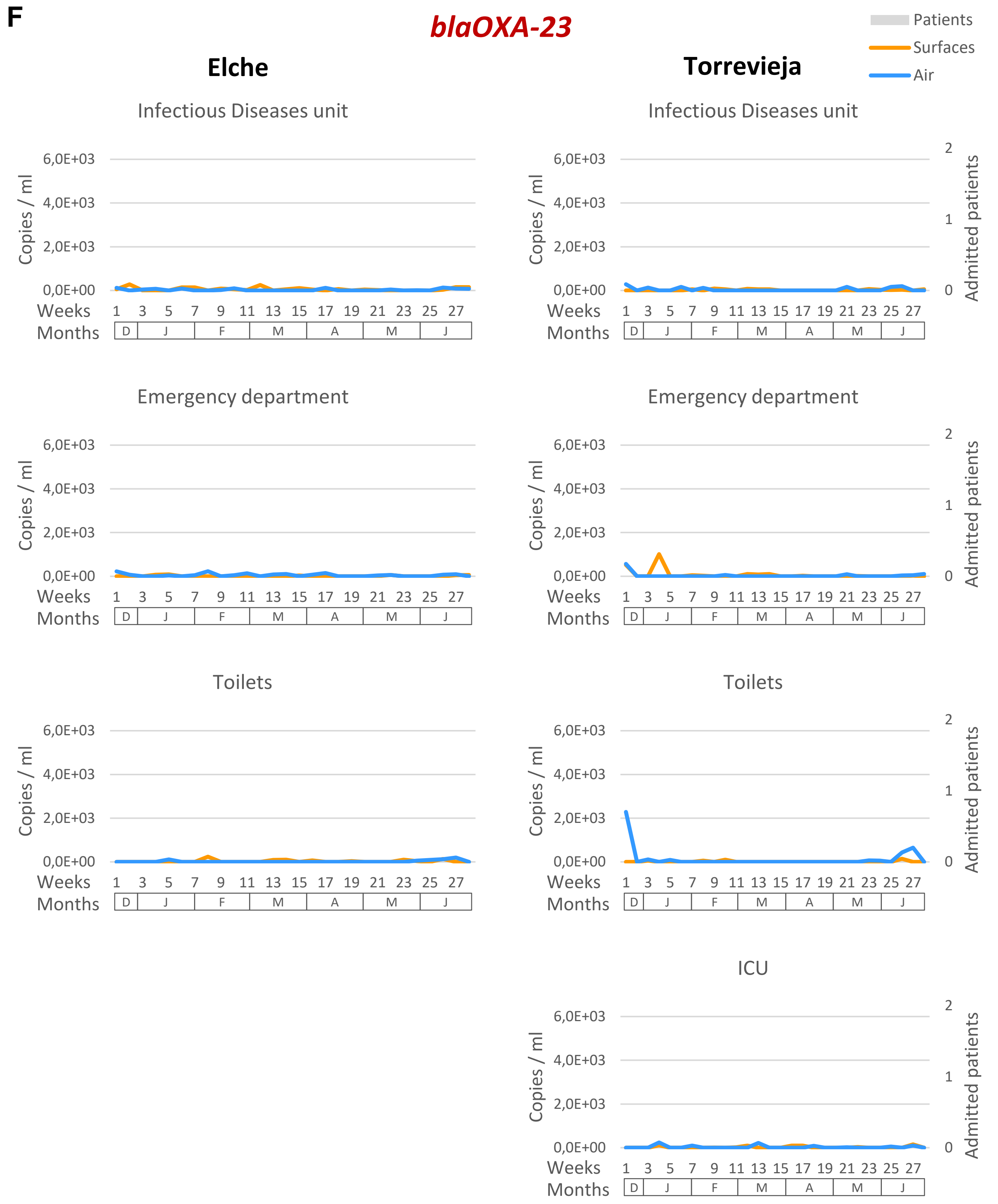
F
blaOXA-23
Patients
Surfaces
Air
Elche
Torrevieja
Infectious Diseases unit
Emergency department
Toilets
ICU
Copies / ml
Admitted patients
6,0E+03
4,0E+03
2,0E+03
0,0E+00
2
1
0
Weeks
1 3 5 7 9 11 13 15 17 19 21 23 25 27
Months
D J F M A M J

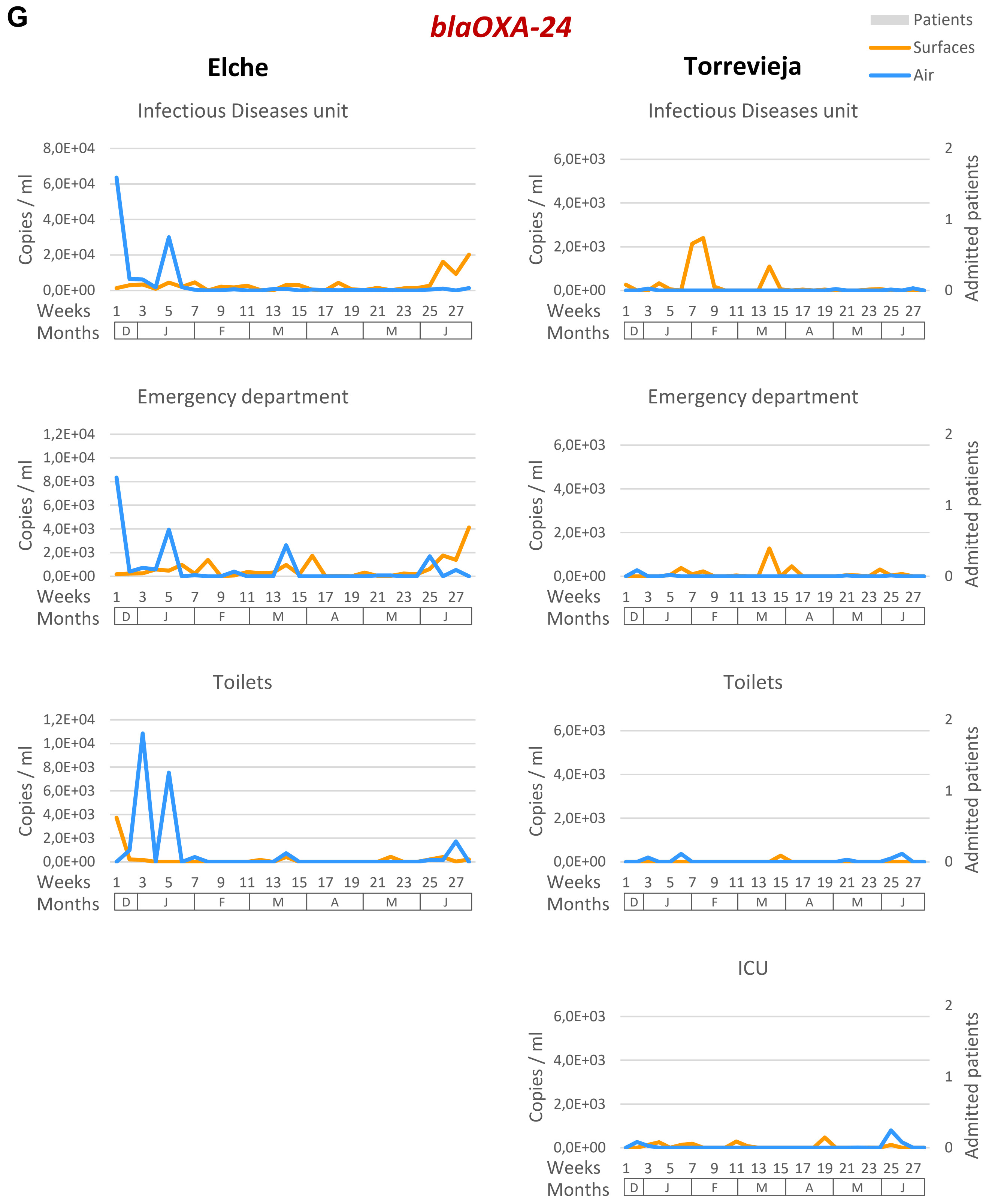
G
blaOXA-24
Patients
Surfaces
Air
Elche
Torrevieja
Infectious Diseases unit
Emergency department
Toilets
ICU
Copies / ml
Admitted patients
Weeks
Months
D J F M A M J

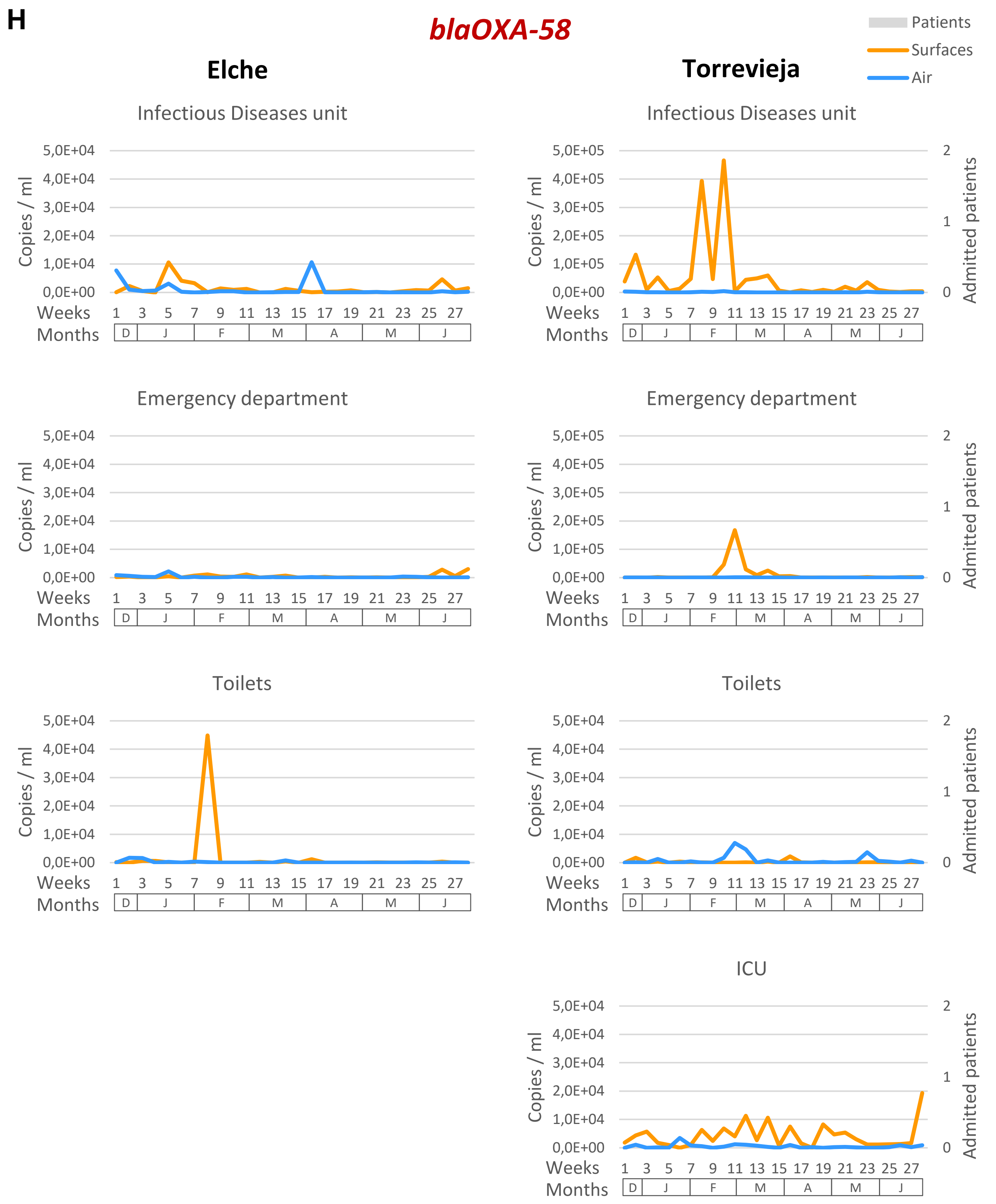
H
blaOXA-58
Patients
Surfaces
Air
Elche
Torrevieja
Infectious Diseases unit
Emergency department
Toilets
ICU
Copies / ml
Admitted patients
5,0E+04
4,0E+04
3,0E+04
2,0E+04
1,0E+04
0,0E+00
5,0E+05
4,0E+05
3,0E+05
2,0E+05
1,0E+05
2
1
0
Weeks
1 3 5 7 9 11 13 15 17 19 21 23 25 27
Months
D J F M A M J

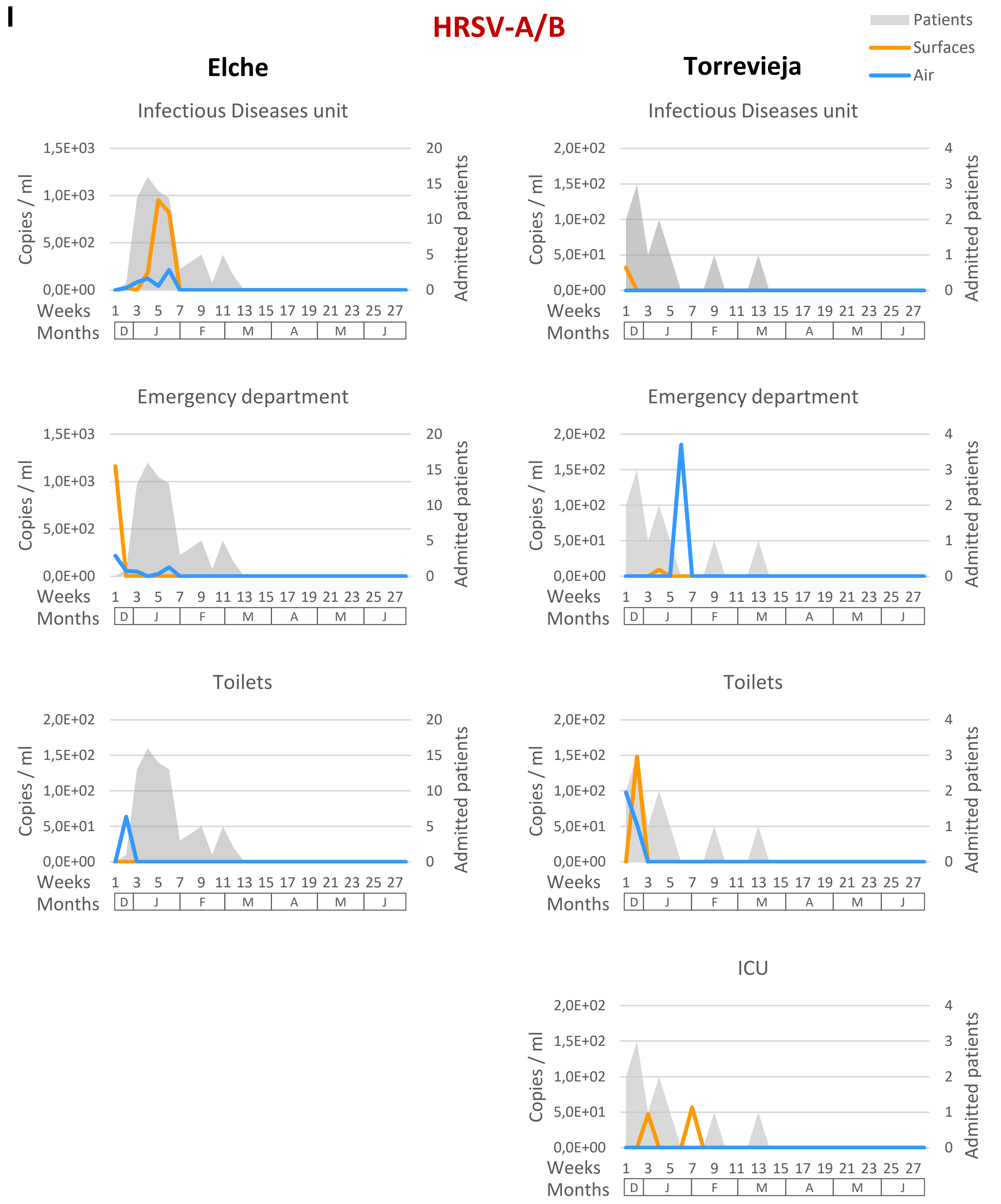
I
HRSV-A/B
Patients
Surfaces
Air
Elche
Torrevieja
Infectious Diseases unit
Emergency department
Toilets
ICU
Copies / ml
Admitted patients
1,5E+03
1,0E+03
5,0E+02
0,0E+00
2,0E+02
1,5E+02
1,0E+02
5,0E+01
Weeks
1 3 5 7 9 11 13 15 17 19 21 23 25 27
Months
D J F M A M J

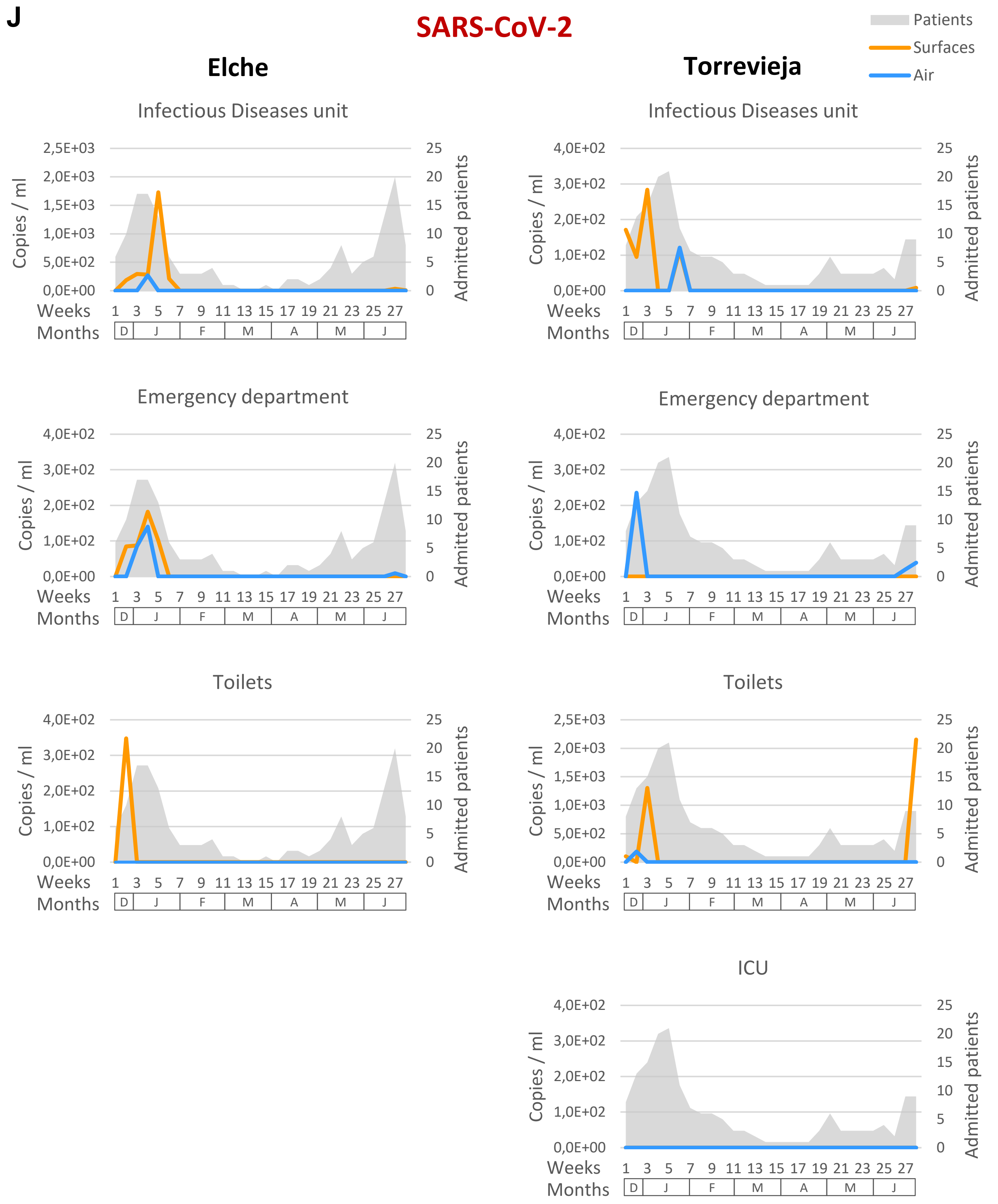

J
SARS-CoV-2
Patients
Surfaces
Air
Elche
Torrevieja
Infectious Diseases unit
Emergency department
Toilets
ICU
Copies / ml
Admitted patients
Weeks
Months
D J F M A M J
